# Prevalence and Risk Factors for Congenital Toxoplasmosis in Newborns in the Public Health System in the Eastern Region of the Brazilian Amazon, Northern Tocantins State, Brazil: Retrospective Cohort Study

**DOI:** 10.3390/tropicalmed11010013

**Published:** 2025-12-31

**Authors:** Stela B. C. Sousa, Cláudia D. M. Mangueira, Sandro E. Moron, Raphael G. Ferreira, Helierson Gomes, Noé M. E. P. L. Costa, Alex S. R. Cangussu, Bergmann M. Ribeiro, Fabricio S. Campos, Gil R. dos Santos, Raimundo W. S. Aguiar, Kelly M. I. Silva, Alice R. Mazutti, Julliana D. Pinheiro, Frederico Eugênio, Erica E. L. Gontijo, Sara F. de Sousa, Jaqueline C. M. Borges, João B. Neto, Marcos G. da Silva

**Affiliations:** 1 Medical School, Health Sciences Center, Federal University of Northern Tocantins (UFNT), Araguaína 77814-350, TO, Brazil; stela.correa@mail.ufnt.edu.br (S.B.C.S.); claudia.denise@mail.ufnt.edu.br (C.D.M.M.); sandromoron@mail.ufnt.edu.br (S.E.M.); ovraphael@gmail.com (R.G.F.); helierson.gomes@ufnt.edu.br (H.G.); 2 Graduate Program in Biotechnology, Federal University of Tocantins, Gurupi 77402-970, TO, Brazil; noe.eiterer@mail.uft.edu.br (N.M.E.P.L.C.); alexcangussu@uft.edu.br (A.S.R.C.); bergmannribeiro@gmail.com (B.M.R.); camposvet@gmail.com (F.S.C.); gilrsan@uft.edu.br (G.R.d.S.); rwsa@mail.uft.edu.br (R.W.S.A.); kelly.farma@outlook.com (K.M.I.S.); alicemazutti00@gmail.com (A.R.M.); 3 Gurupi Regional Hospital, Gurupi 77405-110, TO, Brazil; jullianapinheiro@unirg.edu.br (J.D.P.); fredgpi@hotmail.com (F.E.); 4 Graduate Program in Biosciences and Health at UNIRG, Gurupi 77425-500, TO, Brazil; ericagontijo@unirg.edu.br (E.E.L.G.); sarafalcao@unirg.edu.br (S.F.d.S.); jaquelinecibene@unirg.edu.br (J.C.M.B.); joao@unirg.edu.br (J.B.N.)

**Keywords:** congenital toxoplasmosis, risk factors, vertical transmission, retrospective cohort study

## Abstract

Objective: To determine the prevalence of and risk factors for congenital toxoplasmosis in neonates treated in the public health network of the eastern region of the Brazilian Amazon, northern Tocantins state. Methods: A retrospective cohort study was conducted with neonates born to mothers with gestational toxoplasmosis who received care between 2017 and 2024. The outcome under analysis was positivity for immunoglobulin M in the electrochemiluminescence assay (CLIA). We estimated the prevalence of transplacental infection and respective 95% confidence intervals (95% CI) and its association with risk factors using the odds ratio (or) with a *p*-value < 0.05 in infected neonates before and after 16 gestational weeks at maternal infection diagnosis. Results: A total of 1142 neonates were surveyed, in which 496 were diagnosed with congenital toxoplasmosis (IgM positive), thus obtaining a prevalence of vertical transmission of 45.4%. The main risk factors for vertical transmission were the mother’s education level equal to or less than eight years, (OR = 1.5; 95% CI 1.2; 2.0) and having less than six prenatal consultations (OR = 22.8; 95% CI 3.0; 172.6). Conclusions: A high prevalence of congenital toxoplasmosis was observed, with higher rates of infection in neonates born to mothers with lower levels of education.

## 1. Introduction

Congenital toxoplasmosis is a parasitic disease with high morbidity and mortality among neonates [1,2]. The risk of transmission depends on gestational age and therapeutic intervention at the onset of infection [3,4,5]. Infected fetuses and newborns can suffer serious consequences as a result of infection, such as retinochoroiditis, encephalitis, intracranial calcification, hydrocephalus and death [6].

Adequate prenatal care and early diagnosis, followed by targeted treatment, can prevent vertical transmission and consequently avoid or minimize congenital toxoplasmosis [7].

Despite the high prevalence and possible severity of the outcome, this disease is still a neglected disease [8]. In Brazil, 1183 infant deaths associated with congenital toxoplasmosis were identified between 2000 and 2020. The infant mortality rate associated with congenital toxoplasmosis showed an increasing trend in the country in the years analyzed [9].

Given the importance of this disease, this study aims to determine the prevalence of and risk factors for congenital toxoplasmosis in neonates treated within the public health system in the north of the state of Tocantins, Brazil.

## 2. Materials and Methods

### 2.1. Study Design

The reporting of this study adheres to the “STROBE Statement” (Strengthening the Reporting of Observational Studies in Epidemiology), which consists of 22 items to be included in observational studies [10].

### 2.2. Context

**Figure 1:**
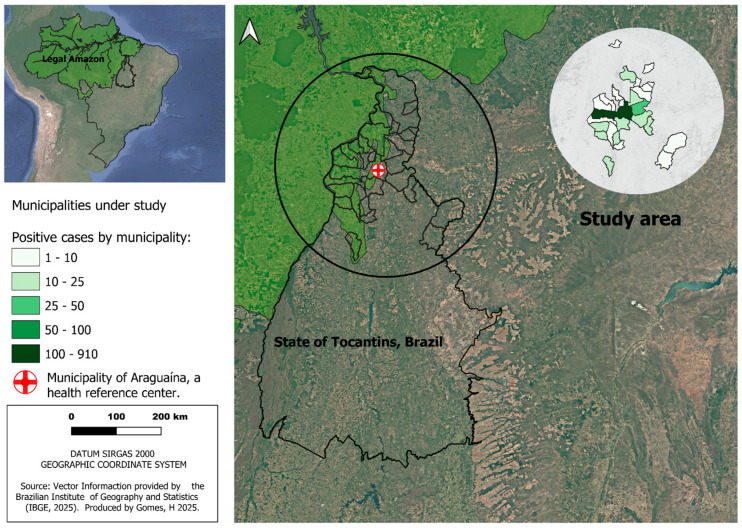
Geographic location of the municipalities of origin of the mothers of newborns with congenital toxoplasmosis who formed the study group, located in the eastern region of the Brazilian Legal Amazon, in the state of Tocantins, Brazil.

This is a retrospective cohort study that includes 1142 neonates born to mothers with gestational toxoplasmosis and treated at the referral hospital of the municipality of Araguaína, the eastern region of the Brazilian Amazon, northern Tocantins state, Brazil, between February 2017 and December 2024. All municipalities studied are located in the northern macro region of the state of Tocantins, in the eastern region of the Brazilian Amazon, in northern Brazil. In total, there are 26 municipalities with a combined population of 370,000 inhabitants [11]. Araguaína, a reference city in the health network, represents 50% of the general population in the study area (Figure 1).

Another characteristic is the large number of small municipalities with internal health structures limited to primary health care, depending on the transfer of more serious patients to other cities when they need more specialized care and a hospital network for medium and high complexity, which in turn are totally dependent on the public health system [11].

Highly complex pregnancy care and deliveries in the study region are referred to the Dom Orione philanthropic hospital, affiliated with the SUS (Brazilian Unified Health System). Located in the municipality of Araguaína, it has around 250 beds and is a major healthcare institution in Tocantins, with ICUs, a surgical center, and various specialties. It is a regional reference and serves both SUS and private health insurance patients. It has a total of 72 obstetric beds. This capacity is divided into 30 surgical obstetrics beds and 42 clinical obstetrics beds. It performs an average of 5500 deliveries per year through the SUS. In 2024, 5517 deliveries were performed throughout the health coverage area [12].

In the region’s public health system, toxoplasmosis screening is part of the prenatal protocol, as recommended by the Ministry of Health. All pregnant women seeking prenatal care undergo initial serological screening for *T. gondii*. Those who present seroconversion or a profile suggestive of acute infection are monitored, and their newborns are included in the criteria of this study, as described. All study participants were SUS patients.

The study was conducted at Dom Orione Hospital, a large tertiary institution with 250 beds, including an emergency room, adult and pediatric wards, and adult and pediatric ICUs. This hospital records an average of 5517 births per year and is the main obstetric and pediatric care center for highly complex cases in the northern region of Tocantins. The hospital’s structure allows for the monitoring and treatment of high-risk pregnant women and newborns, including those with suspected or confirmed congenital toxoplasmosis. Within the scope of this study, all mothers included were tested for toxoplasmosis, according to the inclusion criteria of maternal seropositivity. Testing rates in the private healthcare system were not investigated, as this study focused exclusively on the public healthcare system.

The selection of subjects was based on the seropositivity of their mothers during prenatal care. Pregnant women who presented at prenatal consultations with any detectable anti-*T. gondii* IgM antibodies, or with low-avidity anti-*T. gondii* IgG, as well as those who developed these antibodies during pregnancy (seroconverted), were considered acutely infected, and their neonates were included in the study. The inclusion criteria were maternal anti-*T. gondii* seropositivity confirmed by laboratory tests and prenatal care performed in basic health units in the municipalities of northern Tocantins. Neonates born to chronically infected or seronegative mothers were excluded.

### 2.3. Participants

Only neonates whose IgG and IgM serological tests were adequately performed using the electrochemiluminescence technique were included, totaling 1142 neonates, in accordance with the probabilistic sample calculation.

A 5 mL sample of peripheral blood was collected from each pregnant woman (in the first, second and third trimester of pregnancy) to perform the electrochemiluminescence test to screen for the presence of specific anti-*T. gondii* antibodies of the classes immunoglobulin G and/or immunoglobulin M. In addition, the neonates were subjected to the parasitological and clinical investigation protocol, in which 5 mL of blood was collected on the eighth day of life and examined serologically for the identification of immunoglobulin M and immunoglobulin G.

Positivity for anti-*T. gondii* IgM in the peripheral blood of the newborn collected on the eighth day of life was considered the diagnostic criterion for congenital toxoplasmosis, confirming vertical transmission.

Screening tests were also performed ten days after birth, including the heel-prick test, the red reflex eye test, the hearing test, and the tongue test. They underwent further serology to confirm the presence of congenital infection and identify it in cases in which this diagnosis had not yet been made. The confirmed cases were referred to the at-risk pregnancy service for clinical and laboratory evaluation and appropriate treatment (When maternal infection was discovered up to the 16th week of pregnancy, spiramycin was used as maternal prophylaxis, and when it was discovered after the 16th week of pregnancy, the triple regimen (Pyrimethamine, Sulfadiazine, and Folinic Acid) was used to mitigate the clinical manifestations of the disease). Neonates with clinical indications underwent transfontanelle ultrasound or computed tomography for the diagnosis.

### 2.4. Variables

The variables were categorized as dependent and independent. The dependent variable was a positive IgM serology result in the neonates, while the independent variables included maternal sociodemographic factors (maternal age, years of schooling, residence, race/skin color, city of residence), gestational and clinical factors (gestational age at diagnosis in weeks, number of prenatal consultations, sex of the neonate, maternal treatment with spiramycin, neonatal abnormalities, type of neonatal abnormalities, classification of the neonate according to gestational age, and assessment by the heel-prick test, red reflex eye test, tongue test and hearing test). Prematurity was defined as neonates born before 37 weeks. Low birth weight was defined as a neonate weighing less than 2500 g. The red reflex eye test was used to screen for posterior segment abnormalities and opacities along the visual axis. Pulse oximetry screening was performed at two sites—the right hand and a foot—as a neonatal tool for the early detection of critical congenital heart disease and hypoxemia [13]. The tongue examination assessed the anatomical and functional characteristics of the tongue, allowing for the diagnosis of more severe cases and enabling lingual frenotomy to be performed while the neonate was still in the maternity ward [12]. Ankyloglossia is a condition in which the lingual frenulum is shorter or thicker than normal, limiting tongue mobility. The hearing screening test uses equipment that delivers an auditory stimulus at an intensity of 35 dB and records the result objectively as either “pass” or “fail” to confirm the integrity of cochlear function [14]. The unit “dB” refers to decibels, a measure of sound intensity. In this context, the value indicates the sound level used to assess the infant’s auditory response. If the ear responds to the sound, the test is recorded as a pass; if not, it is considered a fail. This level of sound heard allows the detection of potential hearing impairment.

### 2.5. Data Sources and Measurement

The data were obtained from information recorded in the database of the referral hospital of the municipality of Araguaína, state of Tocantins, Brazil.

### 2.6. Bias Control

To reduce bias, the groups were matched by maternal age and race/skin color with the group of women who had seronegative children (Table 1). To preserve internal validity, the same serological test kit (Cobas e411, Roche, Indianapolis, IN, USA) was used for all pregnant women, and all analyses were processed in a single laboratory to avoid measurement errors, using the electrochemiluminescence method. The tests were processed centrally at the reference laboratory of the Municipality of Araguaína, Tocantins.

### 2.7. Serological Test

The IgG and IgM serological test was performed using the Roche (Elecsys) Cobas e411 kit (Roche Diagnostics GmbH, Mannheim, Germany) and employed the electrochemiluminescence immunoassay (ECLIA) technique, an automated method for the detection and quantification of specific antibodies in serum. The test principle involves incubating samples with *T. gondii* antigens and ruthenium-labeled reagents, whose light emission, activated by an electric current, is directly proportional to the antibody concentration. For IgM antibodies, values expressed in IU/mL below 0.50 were considered non-reactive (Negative), indicating the absence of recent infection; values between 0.50 and 0.60 were indeterminate, requiring re-evaluation; and values greater than or equal to 0.60 were reactive (Positive), suggesting recent infection. For IgG antibodies, which indicate past infection or immunity, a cut-off below 1.6 IU/mL was considered Non-Reactive, and a cutoff greater than or equal to 1.6 IU/mL was considered Reactive.

The IgG anti-*T. gondii* avidity test was performed on the Roche Cobas e411 analyzer, and the results were expressed as a percentage of avidity. Low avidity, below 30–40% binding, suggests a recent infection (within the last 3 to 4 months). High avidity, with values above 60%, indicates an old infection (more than 4 months ago), which can rule out the risk of congenital infection in the current pregnancy if seroconversion occurred previously. Indeterminate results (between 30% and 60%) require further investigation for precise infection dating.

### 2.8. Sample Size

The parameters for the sample size estimation were an expected prevalence of 50%, a confidence level (Type I error) of 5%, and a precision level of 5%. An additional 5% was estimated to account for potential losses. A minimum sample of 444 neonates was defined. Neonates were selected by convenience; those born to mothers who presented for treatment at the reference service were included in the study.

### 2.9. Statistical Analysis

The analysis was conducted using IBM SPSS Statistics software (Version 31.0.0). Variable selection for the multivariate model was performed using a backward stepwise selection procedure. The prevalence of congenital toxoplasmosis, defined by immunoglobulin M (IgM) positivity in neonates, was calculated with its respective 95% confidence intervals (95% CI).

Associations between independent variables (sociodemographic and clinical) and the dependent variable (anti-*T. gondii* IgM positivity) were evaluated using univariate and multivariate analyses, estimating risk through Odds Ratios (OR).

The adjusted Odds Ratio (OR) was calculated by considering maternal gestational age at diagnosis (categorized as ≤16 weeks or >16 weeks) as a moderating variable. This procedure aimed to assess whether the association between risk factors and congenital toxoplasmosis in neonates varied according to this gestational diagnostic window.

Due to the predominantly categorical nature of the independent variables included in the logistic regression models, formal normality tests for predictors were not applicable. All statistical analyses were performed considering a significance level of *p* < 0.05.

## 3. Results

Spatial analysis of confirmed cases of congenital toxoplasmosis and their demographic indicators revealed a heterogeneous distribution among municipalities in the northern region of the state of Tocantins (Figure 1). A significant concentration of absolute cases and a larger population contingent was observed in the municipality of Araguaína, which stood out as the main demographic and healthcare hub in the region. However, when considering incidence, some municipalities with smaller populations had proportionally higher rates, indicating a higher relative risk regardless of population size.

**Figure 2:**
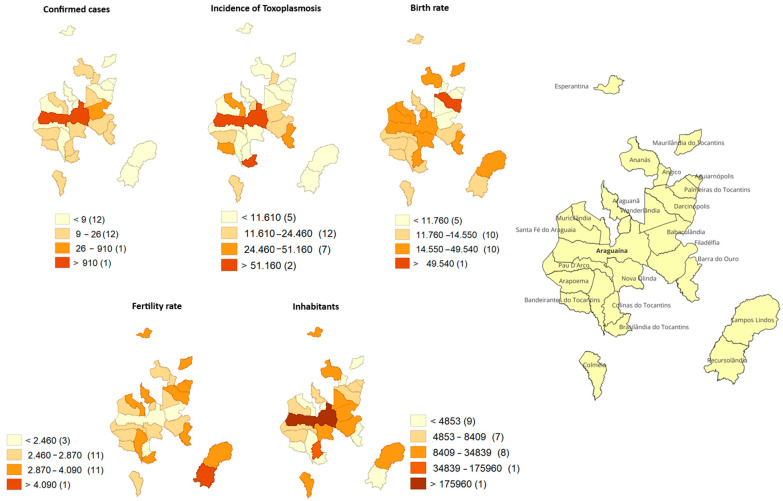
Distribution of cases, incidence of congenital toxoplasmosis, birth rate per year, fertility rate, and number of inhabitants by municipality of origin of mothers of newborns with congenital toxoplasmosis who comprised the study group, located in the eastern region of the Brazilian Amazon, in the state of Tocantins, Brazil.

Birth and fertility rates showed distinct and non-coinciding spatial patterns, suggesting heterogeneous reproductive profiles among the municipalities analyzed. Integrated thematic map overlays show that the burden of congenital toxoplasmosis is not evenly distributed across the territory, being simultaneously influenced by population, demographic, and social factors, which reinforces the need for epidemiological surveillance strategies and the organization of maternal and child care networks guided by territorial criteria (Figure 2).

**Figure 3:**
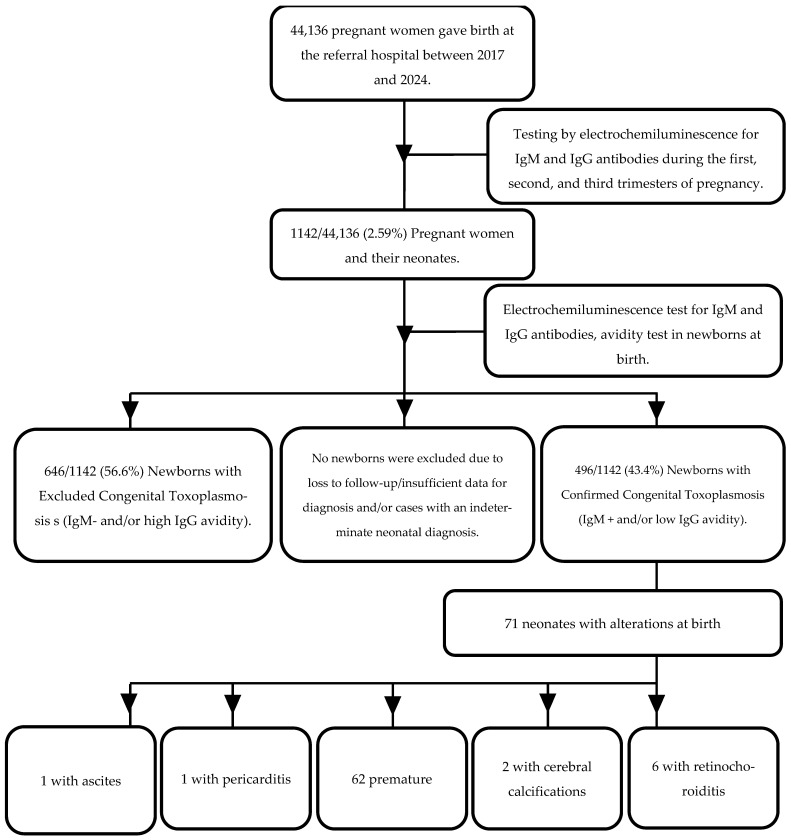
Flowchart of screening in 44,136 pregnant women and selection of 1142 women and their newborns tested by chemiluminescence immunoassay (CLIA) for IgM and IgG antibodies against Toxoplasma gondii, and clinical characteristics of infected newborns with anomalies at birth.

Between February 2017 and December 2024, 1142 neonates were evaluated (Figure 3) at eight days of life; all were born to mothers with gestational toxoplasmosis confirmed by serology.

All the participants underwent anti-*T. gondii* serological testing, and 496 (43.4%) neonates were diagnosed with congenital toxoplasmosis, determined by the presence of IgM antibodies in peripheral circulation, indicative of vertical transmission.

Of the 496 neonates with confirmed congenital toxoplasmosis, 422 (85%) exhibited low IgG avidity, indicative of recent infection. The remaining cases, 74 (15%), showed indeterminate avidity, with no occurrence of high avidity observed. This avidity profile reinforces the diagnosis of vertical transmission of acute infection.

Regarding maternal sociodemographic risk factors for vertical transmission, low maternal education and fewer than six prenatal consultations were associated with transmission of toxoplasmosis to the neonates. In the analysis stratified by gestational age at diagnosis (<16 weeks), only maternal education remained significant in both strata (Table 2). The vertical transmission rate after 16 weeks of gestation was 11 times higher (OR = 11.1; 95% CI: 5.8–21.1) than before this period; however, the severity or presence of fetal abnormalities did not differ between neonates diagnosed before or after 16 weeks. Maternal residence, neonatal sex, maternal age and maternal race/skin color were not significantly associated with seroreactivity in the neonates.

Regarding maternal clinical risk factors for vertical transmission, the univariate analysis showed that maternal treatment with spiramycin, neonatal abnormalities and small-for-gestational-age neonates were associated with the transmission of toxoplasmosis to the infants. However, in the multivariate analysis, no clinical risk factors were significantly associated with transmission in neonates whose mothers were diagnosed before 16 weeks of gestation (Table 3).

Any information contained in the tables can be checked in the spreadsheet containing the total data and is available in the Supplementary Material for the article.

## 4. Discussion

It is essential to note that, for the purposes of this study, “confirmed congenital infection” was defined by the presence of anti-*T. gondii* IgM in newborns on the eighth day of life, which reflects active vertical transmission detectable in the immediate neonatal period, and that longitudinal monitoring of IgG would be necessary to confirm all cases of congenital infection [15].

The results demonstrate a high rate of vertical transmission in the studied population, highlighting the significant impact of this disease on the local neonatal population. Although this prevalence is elevated, it may still be underestimated, as the infection can be detected later due to neonatal immaturity [16]. This finding represents a challenge for healthcare services, both in terms of prevention and in minimizing neonatal sequelae resulting from parasitic effects on the tissues of neonates.

One limitation of this study was that fetal seropositivity was assessed only on the eighth day after birth. It is possible that some neonates were infected but tested negative, as high maternal IgG antibody titers can mask the binding sites of the neonate’s IgM antibodies, resulting in false-negative IgM results. Additional cases of seropositive neonates may emerge later, as well as the possibility of new parasitic lesions [3,6]. Therefore, the results presented are likely conservative and may underestimate the true prevalence over time.

An association was identified between maternal education level and the risk of transplacental transmission of toxoplasmosis, with mothers with less than eight years of schooling showing a 1.7-fold higher likelihood of having an infected neonate. This finding is consistent with the literature, which classifies congenital toxoplasmosis as a neglected disease associated with poverty and low levels of maternal education [17,18,19,20].

Another finding that may partly explain the high prevalence of vertical transmission in the group was the frequency of prenatal consultations, since about 3.4% (17/496) of the pregnant women with infected neonates underwent less than 6 prenatal consultations; in contrast, only 0.1% (1/646) of the group of women with seronegative neonates had less than 6 medical consultations. This indicates that inadequate prenatal care may have influenced the prevalence of infection in the studied group. This finding is consistent with studies conducted in different regions of Brazil and worldwide [1,7,21,22,23]. Prenatal consultations are the key point for greater knowledge of congenital diseases such as toxoplasmosis and, according to the Brazilian Ministry of Health, at least 6 consultations are necessary during pregnancy [24].

Similar results were found in a university hospital in the city of Cuiabá, in the state of Mato Grosso. The cross-sectional study involved 140 pregnant women infected by toxoplasmosis, and 67.9% had had six or more prenatal consultations, followed by 32.1% with less than 6 consultations, in which a correlation was observed between the number of prenatal consultations and vertical transmission of the protozoan [25].

Definitive diagnosis is essential in the first gestational trimester, a phase in which the risk of vertical transmission of toxoplasmosis is not as high as in the final trimester, contrary to the severity of the congenital disease, which is greater at the beginning of pregnancy [26]. Early diagnosis and treatment with spiramycin would help mainly in the prevention of fetal involvement.

We found a direct relationship between the frequency of infection and gestational age at diagnosis, with most women acquiring the infection during the second and third trimesters. This finding is consistent with the majority of studies on the subject, which demonstrate a direct relationship between gestational age at diagnosis and the prevalence of congenital toxoplasmosis: the earlier in pregnancy the infection occurs, the lower the likelihood of disease, whereas the later in gestation, the higher the likelihood of transmission [22]. However, the interpretation of gestational age in relation to congenital toxoplasmosis is not limited solely to the risk of transmission; it also encompasses the severity of lesions and the therapeutic strategy adopted. When it occurs in the first trimester of pregnancy, congenital toxoplasmosis has a greater potential for tissue damage and injury to neonatal tissues due to fetal immaturity [5].

In maternal infections diagnosed before 16 weeks, despite the statistically lower risk of vertical transmission, fetal vulnerability is maximal due to intense organogenesis and neurogenesis. Successful transmission during this period can result in devastating and irreversible fetal lesions, such as hydrocephalus or extensive cerebral calcifications. Therapeutic management with spiramycin at this stage predominantly aims at preventing fetal infection.

On the other hand, maternal infections diagnosed after 16 weeks present a significantly higher risk of vertical transmission. Nevertheless, the fetus already has more developed organs, which may lead to less severe clinical manifestations at birth, or even asymptomatic ones, although the risk of late sequelae, such as chorioretinitis, remains. In these cases, triple therapy (pyrimethamine, sulfadiazine, and folinic acid) is indicated to treat the fetal infection and mitigate its consequences.

Therefore, the use of gestational age at maternal diagnosis as a moderating variable in our multivariate logistic regression models allowed us to evaluate how the impact of risk factors and the effectiveness of therapeutic interventions may vary within these critical developmental windows. This approach offers valuable insights into the disease dynamics and the effectiveness of management strategies based on the clinical timeline of detection and treatment.

Early treatment with spiramycin was not administered in 14 cases (2.8%) in which neonatal seroconversion occurred, compared to one case (0.1%) among seronegative neonates (OR = 18.7; 95% CI: 2.5–143.0), highlighting the importance of appropriate treatment as a protective factor against vertical transmission [27].

Neonatal screening is based on performing laboratory tests during the first days of life, allowing treatment to begin within a time window in which it is possible to prevent or reduce developmental sequelae in the newborn [22].

We observed that, of the 1127 pregnant women who used spiramycin during pregnancy, 482 transmitted the disease to their children (42.77%). However, among the 15 mothers who did not use spiramycin, 14 transmitted the parasite to their children (93.33%), thus demonstrating the significant protective power of spiramycin in preventing transplacental transmission.

However, of the 71 newborns with lesions, 57 (80.28%) underwent complete and adequate treatment with spiramycin and/or the triple regimen of sulfadiazine, pyrimethamine, and folic acid during pregnancy. This information indicates that there are still factors to be explored in the prevention and treatment of this infection. One of the probable causes of these lesions in treated newborns may be the presence of strains that are resistant to treatment or highly virulent.

All the pregnant women with an acute condition, including suspected cases awaiting serological confirmation, should receive prophylaxis from the moment that the possibility of acute infection was determined until the 16th week of gestation and from then on, until delivery, the triple regimen with pyrimethamine, sulfadiazine and folic acid should be used [28].

These data denote the importance of the protocol of the Brazilian Ministry of Health, which advocates treatment with spiramycin early in the first trimester of pregnancy [29]. The treatment should begin with the use of spiramycin shortly after the diagnosis of maternal infection in the first trimester of pregnancy [30]. In cases of fetal abnormalities, treatment should be switched to sulfadiazine combined with pyrimethamine, since spiramycin does not cross the placental barrier. In addition, it is recommended that folinic acid be added to the regimen to minimize the adverse effects of pyrimethamine, particularly bone marrow suppression [31].

Pregnant women who do not undergo treatment are 8.6 times more likely to have an unfavorable outcome, such as fetal death, compared to those who undergo complete treatment [32].

The treatment of infected neonates is mandatory and should continue throughout the first year of life, even in asymptomatic cases, using the therapeutic regimen with sulfadiazine, pyrimethamine and folinic acid according to the Brazilian Ministry of Health [33]. Specific treatment for toxoplasmosis in infected neonates can reduce ocular and neurological sequelae when compared to those who did not undergo the correct treatment [34,35].

Neonates with toxoplasmosis had more than a 110-fold increased risk of presenting abnormalities at birth (OR = 110.1; 95% CI: 15.2–795.4). No neonatal abnormalities were observed in neonates diagnosed before 16 weeks of gestation, which is consistent with the literature. However, this does not preclude the possibility of later reactivation of the infection and delayed damage [7,36,37,38].

In this study, a high number of neonates (14.3%) were born with some form of alteration, the most frequent being prematurity, retinochoroiditis and cerebral calcification, a finding similar to other studies that report that the clinical spectrum of the newborn can range from asymptomatic to more serious sequelae such as brain damage and fetal death [33,39].

The most prevalent clinical manifestations include alterations in the reticulo-endothelial system such as hepatomegaly and splenomegaly, ascites, lymphadenopathy, prematurity, thrombocytopenia and anemia, low birth weight, microcephaly or hydrocephalus, strabismus, convulsive seizures, encephalitis, blindness, deafness and developmental delay [28].

Data on 232 neonates with congenital toxoplasmosis at the obstetric inpatient unit of the Hospital de Clínicas de Porto Alegre showed that 29 (12.5%) were premature and 23 (9.9%) had low birth weight [38]. In this study, similar numbers were found, since 22 babies (4.4%) of the selected sample had low birth weight. In 2015, Fochi observed a 25% prevalence of prematurity and low birth weight in neonates with congenital toxoplasmosis in the state of São Paulo [37], which is concordant with what was found in our study.

Regarding ocular lesions, retinochoroiditis and abnormalities detected by the red reflex test were associated with congenital toxoplasmosis, consistent with most studies implicating *T. gondii* as a cause of ocular damage.

One interesting finding in this study was the association between ankyloglossia and toxoplasmosis, with three cases being observed. A possible explanation is that ankyloglossia may be related to prematurity rather than directly to toxoplasmic infection [40,41,42].

For future investigations or in specific clinical contexts, the incorporation of anti-Toxoplasma IgA analysis could further refine the diagnosis, especially in neonates with indeterminate or negative IgM results but strong clinical suspicion.

## 5. Conclusions

Overall, the results demonstrated a high risk of congenital toxoplasmosis infection and associated lesions in the study population. This risk could be reduced through improved education among at-risk populations and the provision of more effective and widely accessible prenatal care throughout the northern region of the eastern region of the Brazilian Amazon, northern Tocantins state, Brazil.

## Figures and Tables

**Table 1:** Matching of groups of women by age and race skin color of the referral service in Araguaina, the eastern region of the Brazilian Amazon, northern Tocantins state, Brazil, 2025.

Epidemiological Factors	Total	Newborn IgM+		Newborn IgM−		*p*
		*n*	%	*n*	%	
Maternal age						
<30	1016	444	43.70%	572	56.30%	0.603
>30	126	52	41.27%	74	58.73%	
Maternal race/skin color						
White	103	39	37.86%	64	62.14%	0.123
Brown	852	374	43.90%	478	56.10%	
Yellow	22	10	45.45%	12	54.55%	
Black	103	53	51.46%	50	48.54%	
Indigenous	62	20	32.26%	42	67.74%	

*n*: number, %: percentage, IGM: Immunoglobulin M, *p*: Significance level.

**Table 2:** Analysis of crude and adjusted odds ratio (OR) and 95% confidence intervals (95% CI) of sociodemographic characteristics related to congenital toxoplasmosis in 1142 neonates of mothers seropositive for *Toxoplasma gondii* in the referral service in Araguaina, the eastern region of the Brazilian Amazon, northern Tocantins state, Brazil, 2025.

Epidemiological Factors	Number (Positive/Total)	Univariate Model	Multivariate Model
		OR Gross CI 95%	OR Adjusted CI 95%
Maternal age			
<30	444/1.016	1.00	1.00
≥30	52/126	1.05 (0.72; 1.54)	0.90 (0.62; 1.32)
Level of maternal education (years)			
≤8	351/730	1.70 (1.32; 2.19)	1.53 (1.18; 1.99)
>8	145/412	1.00	1.00
Area of mother’s residence			
Rural area	8/22	1.54 (0.59; 4.04)	1.35 (0.56; 3.25)
Urban area	488/1.120	1.00	1.00
Maternal race/skin color			
White	39/103	1.00	1.00
Brown	374/852	1.18 (0.77; 1.83)	0.78 (0.51; 1.19)
Yellow	10/22	1.48 (0.78; 3.77)	0.73 (0.29; 1.85)
Black	53/103	1.41 (0.79; 2.54)	0.57 (0.33; 1.00)
Indigenous	20/62	0.77 (0.38; 1.56)	1.28 (0.66; 2.49)
City of the mother			
Araguaína	386/910	0.89 (0.65; 1.21)	1.22 (0.92; 1.63)
Other cities	110/232	1.00	1.00
Number of prenatal appointments			
<6	17/18	22.89 (3.04; 172.61)	2.97 (0.33; 24.66)
≥6	479/1.124	1.00	1.00
Sex of the newborn			
Female	234/566	1.07 (0.84; 1.37)	1.18 (0.94; 1.50)
Male	262/576	1.00	1.00

OR: odds ratio, CI: confidence interval.

**Table 3:** Analysis of the crude and adjusted odds ratio (OR) and 95% confidence intervals (95% CI) of the clinical characteristics related to congenital toxoplasmosis in 1142 neonates of mothers seropositive for *Toxoplasma gondii* in the referral service in Araguaina, the eastern region of the Brazilian Amazon, northern Tocantins state, Brazil, 2025.

Epidemiological Factors	Number (Positive/Total)	Univariate Model	Multivariate Model
		OR Gross CI 95%	OR Adjusted CI 95%
Gestational treatment with spiramycin			
Yes	482/1.127	1.00	1.00
No	14/15	18.73 (2.45; 142.96)	2.33 (0.28; 19.57)
Gestational phase of spiramycin use			
<16 Weeks	1050/1127	1.00	1.00
>16 Weeks	77/1127	-	-
Alterations in the newborn			
Yes	71/72	110.08 (15.24; 795.40)	2.8 (0.26; 20.08)
No	416/1070	1.00	1.00
Type of alterations in the newborn with congenital toxoplasmosis at birth			
Ascites	1/1	-	-
Pericarditis	1/1	-	-
Prematurity	62/69	1.31 (0.15; 11.05)	3.19 (0.35; 29.44)
Prematurity and brain calcifications	1/1	-	-
Prematurity and retinochoroiditis	1/1	-	-
Retinochoroiditis	5/5	-	-
Classification of the newborn according to gestational age			
Adequate	474/1.116	1.00	1.00
Small	22/26	7.45 (2.55; 21.76)	7.71 (0.90; 66.26)
Large	0	-	-
Red reflex eye test			
Reflex absent in one of the eyes	6/7	7.91 (0.95; 65.92)	1.53 (0.98; 5.56)
Bilateral reflex present	490/1.135	1.00	1.00
Heel-prick test			
Altered	0	-	-
Normal	496/1.142	1.00	1.00
Tongue test			
Ankyloglossia	3/4	3.93 (0.41; 37.91)	-
Normal	493/1.038	1.00	1.00
Hearing screening test			
Altered	0	-	-
Normal	496/1.142	1.00	1.00

OR: odds ratio, CI: confidence interval.

## Data Availability

The data can be accessed directly from the corresponding author.

## Supplementary Materials

The following supporting information can be downloaded at: https://www.mdpi.com/article/10.3390/tropicalmed11010013/s1, Table S1: Global data on mothers and newborns.
